# Household out-of-pocket medical expenditures and national health insurance in Taiwan: income and regional inequality

**DOI:** 10.1186/1472-6963-5-60

**Published:** 2005-09-02

**Authors:** Tu-Bin Chu, Tsai-Ching Liu, Chin-Shyan Chen, Yi-Wen Tsai, Wen-Ta Chiu

**Affiliations:** 1 Taipei Municipal Wan Fang Hospital, Taipei, Taiwan; 2 Department of Public Finance, National Taipei University, Taipei, Taiwan; 3 Department of Economics, National Taipei University, Taipei, Taiwan; 4 Division of Health Plicy Research, National Health Research Institues, Taipei, Taiwan

## Abstract

**Background:**

Unequal geographical distribution of medical care resources and insufficient healthcare coverage have been two long-standing problems with Taiwan's public health system. The implementation of National Health Insurance (NHI) attempted to mitigate the inequality in health care use. This study examines the degree to which Taiwan's National Health Insurance (NHI) has reduced out-of-pocket medical expenditures in households in different regions and varying levels of income.

**Methods:**

Data used in this study were drawn from the 1994 and 1996 Surveys of Family Income and Expenditure. We pooled the data from 1994 and 1996 and included a year dummy variable (NHI), equal to 1 if the household data came from 1996 in order to assess the impact of NHI on household out-of-pocket medical care expenditures shortly after its implementation in 1995.

**Results:**

An individual who was older, female, married, unemployed, better educated, richer, head of a larger family household, or living in the central and eastern areas was more likely to have greater household out-of-pocket medical expenditures. NHI was found to have effectively reduced household out-of-pocket medical expenditures by 23.08%, particularly for more affluent households. With the implementation of NHI, lower and middle income quintiles had smaller decreases in out-of-pocket medical expenditure. NHI was also found to have reduced household out-of-pocket medical expenditures more for households in eastern Taiwan.

**Conclusion:**

Although NHI was established to create free medical care for all, further effort is needed to reduce the medical costs for certain disadvantaged groups, particularly the poor and aborigines, if equality is to be achieved.

## Background

In 2000, Taiwan was ranked as the second healthiest country in the world, only behind Sweden [1]. While its economic growth, environmental sanitation, and health education have been contributing factors, its implementation of National Health Insurance (NHI) in 1995 is the most important key to its achievements in health. With the comprehensive medical benefit coverage provided to almost all people, NHI has notably increased the availability and accessibility of medical care services and has seen a significant increase in utilization of its medical healthcare services [2-5]. Starting with a national approval rate of 33% in 1995, NHI's approval rating rose to 75.4% in 2000 and to over 80% in 2001 [6].

Although many studies examining whether NHI mitigates the inequality in health care utilization have been conducted and have provided solid evidence that NHI does lessen the socio-demographic and regional disparity, few studies have focused on testing how equitable the financing scheme of Taiwan health care system is. Neither have the changes in relative out-of-pocket expenditures during the pre- and post-NHI period been compared. Since a national insurance program should spread risks over large population groups and reduce medical costs for all patients, particularly the poor, it is important to understand how the health care reform (NHI) reduces and to what extent it reduces out-of-pocket expenditures for medical care. The financial burden from out-of-pocket medical expenditures is regressive financing in developed countries such as USA [7] or in developing countries such as Thailand [8], where financial burden is heavier for the poor. That is, the poor pay a larger share of their income for out-of-pocket medical expenditures than the rich in most countries; only a few, including Colombia and Vietnam, have implemented healthcare reforms capable of remedying regressivity and reducing medical costs for the poor [9,10]. No studies, however, have assessed the impact of Taiwan's NHI on out-of-pocket medical expenditures.

Yeh [11]examined the distribution of out-of-pocket medical expenditures among households under Taiwan's NHI and, like most international studies, found regressivity in out-of-pocket financing scheme of NHI, but that study did not evaluate how or if regressivity especially evolved from implementing NHI. One comparative study of pre- and post-NHI household out-of-pocket medical expenditures [12] reported that NHI reduced the gap between the richer and the poor. That study found household out-of-pocket expenditures of those household with incomes in the highest quintile was 2.64 times that of those in the lowest quintile before NHI was implemented, and that this figure was reduced to 2.30 after NHI was implemented. That study, however, did not delineate the differences in out-of-pocket medical expenditures incurred by different income level of households under NHI. In an effort to resolve these unanswered research questions, we examined the direct and indirect impact of NHI on household out-of-pocket medical expenditures in Taiwan by pooling the 1994 and 1996 cohorts of survey data. Our analysis particularly examines the impact of NHI on reducing financial burdens and regional differences in medical care expenditures.

### Public health system in Taiwan

Unequal geographical distribution of medical care resources and insufficient healthcare coverage has been two long-standing problems with Taiwan's public health system.

#### Regional inequality

Two-thirds of Taiwan's terrain is mountainous, and many of these mountainous areas are isolated and inaccessible. Since most of the high mountains are located in the central eastern regions, the eastern coast area is the least developed and populated area in Taiwan. This area, therefore, is lacking in medical manpower and facilities. Taiwan's northern area, however, is its most developed region. It includes Taiwan's largest city, the capital, Taipei. Most medical care facilities and medical personnel are concentrated in such urban areas. This disparity in accessibility to health care services has raised many concerns and complaints from the public.

In 1985, the Medical Care Network project, established to balance the distribution of medical care resources in various regions and to allow medical manpower and facilities to grow at a reasonable rate, divided Taiwan into 17 medical care regions and aimed to develop medical manpower and facilities. The project had three chronological phases: July 1985 – June 1990, July 1990 – December 1996 and January 1997 – December 2000. The second phase was specifically designed to even out differences in the regional distribution of medical care resources in preparation for the implementation of NHI in 1995.

To reach this objective, the Medical Care Act prohibited establishment or expansion of hospitals in areas with excess medical care resources and encouraged their development in areas with fewer resources. A Medical Care Development Fund was established to provide the private sector with financial incentives (subsidizing the interest on loans) to construct medical care institutions in areas with fewer medical resources [13].

#### Insufficient health insurance

In the 1980's, around 40% of the population had no health insurance. The uninsured poor faced financial barriers to health care services and their condition of their health was negatively impacted. Industrialization, urbanization and the aging of population, however, increased people's demands for better health and medical care services. To meet that demand and guarantee all people basic health care use, Taiwan began the arduous task of implementing a national health insurance program [6].

Taiwan had three major social insurance programs before NHI was implemented: Labor Insurance, Government Employees' Insurance and Farmers' Insurance. These programs covered employed workers, who accounted for about 58% of the population. The remaining 42%, approximately 9 million children, elderly people, and non-working adults, had no insurance. The availability and accessibility of medical care services for those formerly insured were not high because services were limited to certain contracted medical care institutions. Those formerly insured by Labor Insurance and Farmers' Insurance received medical care services from contracted medical institutions, which accounted for 80% and 47% of hospitals and clinics, respectively[14,15]. Those formerly insured by Government Employee's Insurance had even fewer choices, with only 61% of the hospitals and less than 6% of clinics contracted by to government to provide medical care for them [16].

NHI integrated the former three social insurance programs into one comprehensive program would also include those previously uninsured. All citizens became eligible for NHI, including legally employed foreigners. The enrollment rate during its inception period in 1995 was 92%, and that figure had increased to 96.16% by 2000. Once NHI was implemented, all beneficiaries were also required to receive health care at contracted medical care facilities, but the availability of such facilities increase significantly. By 1998, they constituted about 93.68% of overall medical facilities in Taiwan [6]

With regard to medical benefit coverage, the three insurance schemes in place prior to NHI only covered some a specified types of medical care services. For example, Labor Insurance and Farmers' Insurance covered three items: ambulatory care, inpatient care, and medical care for child delivery. NHI covered inpatient care, ambulatory care, laboratory and X-ray examinations, prescription drugs and certain OTC drugs, dental services, traditional Chinese medicine, day care for mental illness, limited home care and certain preventive services.

Because the previous insurance schemes required no co-payment, moral hazard is widely believed to occur. To prevent these practices, NHI requests patients to make co-payments for outpatient and inpatient care, dental care, emergency care or Chinese medicine services and pharmaceuticals. Co-payment, however, is not required in certain situations. For example, if a beneficiary suffers a major illness or injury and requires long-term, expensive treatment, he/she is exempted from any co-payment obligation under Article 36 of the National Health Insurance Act. Co-payment exemptions have also been established for childbirth and preventive health services, low-income households, veterans and their dependents and people residing in mountainous areas or on offshore islands.

To prevent the public from incurring catastrophic expenses, NHI has established co-payment ceilings. The co-payment ceiling for expenses incurred within 30 days in an acute ward or within 180 days in a chronic ward was calculated as 6% of the average national income, which came to NT$23,000 per admission in 2001. To further reduce financial burden of patients, the cap on cumulative costs for the calendar year was also set and calculated as 10% of the average national income, which came to NT$39,000 in 2001 [6].

## Methods

### Data

Data used in this study were drawn from the 1994 and 1996 Surveys of Family Income and Expenditure (SFIE) [17,18]. These surveys were conducted by the Directorate-General of the Budget, Accounting and Statistics, Executive Yuan, Republic of China and contained observations for 14,011 and 13,484 households in the pre- and post-NHI period, respectively. The SFIE includes two survey procedures: interviews and account-keeping collection methods. The sampled households, chosen from the population using stratified random sampling, were interviewed once each year to determine their major sources of income and expenditures. Then, to obtain detailed categories of income and expenditure, some of interviewed households were asked to also track daily income and expenditure activity. Because the SFIEs were done by interviews and account-keeping collection methods, they are more accurate than those derived solely from interviews and they have been widely used by domestic and international researchers [19,20].

The data contains detailed information on a series of personal and family characteristics, including age, sex, marital status, highest education level attained, employment status, consumption of health care, individual and household out-of-pocket expenditures, and geographic locations, etc.

### Analytical method

The pre-NHI 1994 survey and the post-NHI 1996 survey enabled us to assess the impact of NHI on household out-of-pocket medical care expenditures shortly after its implementation in 1995. We pooled the data from 1994 and 1996 and included a year dummy variable (NHI), equal to 1 if the household data came from 1996. In addition, the estimated model contains two groups of interaction effects, *Income*_*i*_/*NHI*_*i *_and *Region*_*i*_/*NHI*_*i*_, to examine whether NHI reduced financial barriers and regional differences in household out-of pocket medical expenditures.

The empirical model is specified below:

*Ln*(*Exp*_*i*_) = *β*_0 _+ *β*_1_*Income*_*i *_+ *β*_2_*Region*_*i *_+ *β*_3_*NHI*_*i *_+ *β*_4_*Income*_*i*_/*NHI*_*i *_+ *β*_5_*Region*_*i*_/*NHI*_*i *_+ *β*_6_*Z*_*i *_+ *ε*_*i*_

*Exp*_*i *_is out-of-pocket expenditures on health care by household *i*. For linearity in the equation, the dependent variable (Exp) is transferred into logarithm. *Income*_*i *_is a set of household income dummy variables. *Region*_*i *_is a set of regional dummy variables. *NHI*_*i *_indicates the national health insurance program. *Income*_*i*_/*NHI*_*i *_*and Region*_*i*_/*NHI*_*i *_are two groups of interactions of income and region and NHI. *Z*_*i *_represents a vector of socioeconomic and demographic characteristics of economic household head, which are martial status, sex, employed status, age, education level and family members. The detailed definitions of variables are listed in Table 1.

**Table 1:** Definitions of Variables

Variable	Definition
**Dependent Variable**	
Expenditures	Continuous variable: total household annual out-of-pocket medical expenditures
**Independent Variable**	
Demographic variables	(Economic Household Head, EHH)
Married	Dummy variable = 1 if EHH is currently married, otherwise = 0 including never married, widowed, separated and divorced.
Male	Dummy variable = 1 if EHH is male.
Employed	Dummy variable = 1 if EHH is employed.
Age	
25–44	Dummy variable = 1 if EHH's age in this range.
45–64	Dummy variable = 1 if EHH's age in this range.
65 and above	Dummy variable = 1 if EHH's age in this range. (EHH's age under 25 is the default variable.)
Education	
Junior high	Dummy variable = 1 if EHH finished Junior high school.
Senior (vocational) high	Dummy variable = 1 if EHH finished senior (or vocational) high school.
Junior college	Dummy variable = 1 if EHH finished Junior college.
College and above	Dummy variable = 1 if EHH finished college and above. (Primary school and below is the default variable.)
Family members	Dummy variable = 1 if the number of household members ≥ 4.
NHI	Dummy variable = 1 if EHH is from the post-national health insurance cohort.
Household income	
Lower 20%~2nd quintile	Dummy variable = 1 if family income is lower 20%.
Middle 20%~3rd quintile	Dummy variable = 1 if family income is middle 20%.
Higher 20%~4th quintile	Dummy variable = 1 if family income is higher 20%.
Highest 20%~5th quintile	Dummy variable = 1 if family income is highest 20%. (Lowest 20%~1st quintile is the default variable)
Regional variables	
Center	Dummy variable = 1 if the household is located in: Taichung Hsien, Changhwa Hsien, Nantou Hsien, Yunlin Hsien, Taichung City.
South	Dummy variable = 1 if the household is located in: Chiayi Hsien, Tainan Hsien, Kaohsiung Hsien, Pingtung Hsien, Kaohsiung Municipality, Chiayi City, Tainan City.
East	Dummy variable = 1 if the household is located in: Taitung Hsien, Hwalien Hsien, Penghu Hsien.
North	Dummy variable = 1 if the household is located in: Taipei Hsien, Keelung City, Ilan Hsien, Taoyuan Hsien, Hsinchu Hsien, Miaoli Hsien, and Taipei Municipality. (North is the default variable)

A person in the household who earns the largest personal share of pay in family income is considered to be the economic household head.

### Hypotheses

#### NHI

Prior to NHI, about 40% of the population had no social health insurance. NHI enrolled more than 96%. With the NHI offering generous medical benefit coverage, the medical care for most patients, whose medical expenses had been previous paid out-of-pocket, would be paid by insurance program and their financial burden mitigated. We hypothesized that NHI would reduce out-of-pocket expenditures.

#### Income

Household incomes were categorized into five categories: the lowest 20%, lower 20%, middle 20%, higher 20%, and the highest 20%. The lowest 20% (first quintile) served as our reference group. It has been reported that the higher the income, the greater the utilization of health care and the higher the household out-of-pocket medical expenditures [21]. In other words, the rich would be generally expected to have more out-of-pocket expenditures than the poor. We hypothesized that the coefficient of four dummy income variables would be significant and positive.

#### Income/NHI

Since NHI imposed the ceilings on co-payment to avoid catastrophic expenses, income tended to play a relatively smaller role in access to medical care after NHI. In other words, NHI helps reduce out-of-pocket expenditures for medical care, but the highest difference would be observed in the top income quintile. We expect that the negative impact of NHI would dominate the positive impact of income. Four income/NHI interaction variables were expected to have significant and negative coefficients.

#### Region

As discussed above, uneven distribution of medical care resources in Taiwan has been the target of much criticism. Most resources are concentrated in the North where the population has higher accessibility to health care than residents in non-northern areas (Center, South, East). Out-of-pocket medical expenditures impose a relatively heavier financial burden on residents in non-northern areas than on those in northern areas. The regional factors had four dummy variables, North, Central, South, and East. Therefore, we treated the variable of North as the default variable and hypothesized that the three dummy regional variables would have positive coefficients.

#### Region/NHI

Because out-of-pocket medical expenditures impose a relatively heavier financial burden on residents in non-northern areas than on northern residents, the Bureau of NHI, has, since its inception in 1995, always endeavored to improve medical care accessibility for residents in remote areas. We expect that NHI mitigates regional inequality in out-of-pocket expenditures and hypothesized that the coefficients of region and NHI interactions would be negative.

#### Demographic variables

There were six other groups of variables, which were demographic: martial status, sex, employment status, age, education, and family size. The married household heads are likely to result in more household out-of-pocket medical expenditures than the unmarried due to more health care service use either by them or their spouses. Hence, the marital status variable should have a positive coefficient. Females were found to consume somewhat more health care than males do primarily because of childbearing[22]. In addition, since females are more careful about the health condition of the members in their family and possibly more likely to take them for medical care than males, the female household heads would incur more household out-of-pocket medical expenditures than the male. We expect that the sex variable (Male) should have a negative coefficient. Because it would cost the employed work time to seek health care, they should be less likely to seek care and, therefore, have fewer out-of-pocket medical expenditures than the unemployed. The unemployed, due to depression over being jobless, may also seek more health care then the employed. Being employed/Being unemployed would be expected to have a negative coefficient. Because health often deteriorates as a result of aging, we can assume the older an individual, the more health care he or she will seek. We expect that the three dummy age variables would have a positive impact on out-of-pocket medical expenditures. Most often the higher an individual's education, the more socially advantaged he or she will probably be and the more access he or she will have to medical care. In turn, the more medical care one seeks, the more his or her out-of-pocket medical expenditures. We expected four dummy education variables to have a positive impact. Family size is another important factor in the demand for medical care and the amount of more out-of-pocket medical expenditures. An increase in family size should increase the likelihood of medical care use and result in more out-of-pocket medical expenditures. The family size was expected to have a positive coefficient.

## Results

After the implementation of NHI, total household out-of-pocket medical expenditures dropped from NT$23,046 in 1994 to NT$17,726 in 1996 (23.08%) (Table 3). This drop suggests that NHI effectively reduced household out-of-pocket medical expenditures. The highly significant and negative coefficient of NHI in the empirical model confirms this observation (Table 2).

**Table 2:** Multivariate Regression Results

Variables	Coefficient	Standard Error
Constant	8.6900 ***	0.0604
		
Demographic variables		
Married	0.1511 ***	0.0188
Male	-0.1399 ***	0.0194
Employed	-0.2753 ***	0.0330
Age		
25–44	-0.0284	0.0413
45–64	-0.0158	0.0430
65 and above	0.2983 ***	0.0524
(Under 25 is the default variable)		
Education		
Junior high	0.0561 **	0.0184
Senior (vocational) high	0.1269 ***	0.0169
Junior college	0.2568 ***	0.0220
College and above	0.2891 ***	0.0230
(Primary school and below is the default variable)		
Family members	0.1668 ***	0.0053
		
NHI	-0.2657 ***	0.0391
		
Household income		
Lower 20%~2nd quintile	0.1638 ***	0.0362
Middle 20%~3rd quintile	0.3405 ***	0.0358
Higher 20%~4th quintile	0.5847 ***	0.0363
Highest 20%~5th quintile	0.8981 ***	0.0371
(Lowest 20%~1 quintile is the default variable)		
		
Regional variables		
Center	0.1077 ***	0.0237
South	0.0010	0.0217
East	0.3802 ***	0.0504
(North is the default variable)		
		
Interaction effects		
Lower 20% income/NHI	0.0327	0.0443
Middle 20% income/NHI	-0.0664	0.0441
Higher 20% income/NHI	-0.2465 ***	0.0445
Highest 20% income/NHI	-0.4146 ***	0.0447
Center/NHI	0.0785 **	0.0322
South/NHI	0.1693 ***	0.0286
East/NHI	-0.4471 ***	0.0639
		
Numbers of observation	23925	
F	177.81	
Prob >F	0.0001	
R-squared	0.1621	
Adjust R-squared	0.1612	

*** Significant at 0.01 level; ** significant at 0.05 level; * significant at 0.1 level.

**Table 3:** Mean Differences in Out-of-Pocket Expenditures Before and After NHI

	Pre-NHI (1994)	Post-NHI (1996)	Total percentage change
Total expenditures	23046	17726	-23.08%
North	23885	17495	-26.75%
Center	23503	16950	-27.88%
South	20397	18645	-8.59%
East	29606	17389	-41.27%
			
Family income			
Lowest 20%~1st quintile	13551	11390	-15.95%
Lower 20%~2nd quintile	15218	14559	-4.33%
Middle 20%~3rd quintile	18398	16783	-8.78%
Higher 20%~4th quintile	24565	19409	-20.99%
Highest 20%~5th quintile	35738	26227	-26.61%

Unit: NT$, 1994: 1US$ = 26.24NT$; 1996: 1US$ = 27.79NT$

Table 3 shows that average medical out-of-pocket expenditures increased in conjunction with increased income. Pre-NHI expenditures ranged from NT$13,551 to NT$35,738. Post-NHI expenditures ranged from NT$11,390 to NT$26,227. These statistics reveal that households with income in the higher quintiles seemed to have more out-of-pocket medical expenditures both before and after NHI. That is, income should be positively related to out-of-pocket expenditures in Taiwan. The four household income dummy variables were, as expected, found to have significant and positive coefficients. Moreover, the percentage of expenditures is shown to decrease 15.95% for households in the lowest income quintile after NHI in Table 3. This is less than the decreases in the highest 20% (-26.61%) and higher 20% (-20.99%) income quintiles, but greater than in the lower 20% (-4.33%) and middle 20% (-8.78%) income quintile households. In Table 2, *Income/NHI *interactions were, as expected, found to be significantly related to household out-of-pocket expenditures. All interactions were shown to have negative coefficients except for lower 20% income/NHI. Surprisingly, only two of *Income/NHI *interactions (higher 20% and highest 20%) reached statistical significance.

The range of out-of-pocket expenditures, all four regions included, was $20,397 – $29,606 pre-NHI and $16,950 – $18,645 post-NHI (Table 3). The eastern area had the highest out-of-pocket expenditures before NHI. After NHI, however, the southern area, previously lowest, had the highest out-of-pocket expenditures. Moreover, household out-of-pocket expenditures in the eastern area decreased by 41.27%, a much greater decrease than was found in the northern (-26.75%) and central (-27.88%) areas, and 5 times greater the -8.59% decrease found for the southern area. As can be seen in the summary of the three regional dummy variables in Table 2, the central and eastern areas were found to have significant and positive coefficients. The coefficient for the southern area was statistically insignificant. The coefficients of three *region/NHI *interaction effects were all significant. Only one interaction effect (East/NHI) was negative; Center/NHI and South/NHI were surprisingly found to be positive, suggesting that households in the central and southern areas tended to have more out-of-pocket medical expenditures than those in the northern areas, though NHI had been implemented. Moreover, only households in the eastern area tended to have fewer out-of-pocket medical expenditures than those in the North after NHI.

All demographic variables had significant effects except for two age dummy variables, 25 – 44 and 45 – 64 (Table 2). An individual who was older, female, married, unemployed, better educated or the head of a larger family household was likely to have greater household out-of-pocket medical expenditures. Household heads older than 65 tended to have more out-of-pocket medical expenditures than those below that age. There was no significant difference between household heads younger than 25 years and those between 25 and 64 years old.

## Discussion

According to the Department of Health, total health expenditures increased steadily over the past decade, from 3.96% of GDP in 1981 to 4.93 of GDP in 1994, but jumped up to over 5.27% in 1995 with the introduction of National Health Insurance. During the same period, the annual growth rate of per capita health expenditures averaged below 10%, but reached 15% in 1994/1995, suggesting that there was a considerable increase in health care utilization and health expenditures with the implementation of NHI. [23]

Although NHI program with its generous medical coverage encourages greater utilization of medical care, it also helps to alleviate financial burden of private sector. The figures showed that private expenditures, which constituted 44.71% of total health expenditures in 1993, dramatically decreased from 43.74% in 1994 to 34.57% in 1995.[23] Our study showed a significant decrement in average household out-of-pocket payments from NT$23,046 in 1994 to NT$17,726 in 1996 (23%), further confirming that NHI significantly reduced household out-of-pocket expenditures.

The finding that higher income households are more likely to have larger out-of-pocket medical expenditures than lower income households is consistent with most international studies as indicated above. However, after the implementation of NHI, lower and middle income quintiles had a relatively small decrease in out-of-pocket medical expenditures, suggesting that NHI led to a significant increase in utilization in the lower income groups. The likely explanation may be that since the coverage rate of social health insurance in 1994 was much lower for the two lower income groups than the highest income group (66.7%, 86.3% vs 94.3%), the effect of greater insurance coverage was matched by increasing utilization. Moreover, NHI is found to reduce out-of-pocket medical expenditures more for households with lowest income than those with lower and middle incomes possibly due to the co-payment exemption provided to the lowest-income households. In general, NHI did reduce the gap in out-of-pocket expenditures among different income groups. Pre-NHI household out-of-pocket expenditures in the highest quintile were approximately 3 times more than in the lowest quintile. After NHI, this multiple was reduced to 2.

The households in the central and eastern areas were found to be likely to have more out-of-pocket medical expenditures than households in the northern area. This difference may be because in 1994 health insurance was less prevalent in these two areas than the northern area (84.7%, 82.8% vs 87.6%). Another likely explanation may be that the some parts of central areas and most of eastern areas are remote areas with more mountainous terrain. Therefore, there are fewer medical resources located in these areas, making medical care less accessible there than in the northern area. This inconvenience may discourage utilization and encourage residents to self-medicate, which may negatively affect their health and possibly result in future catastrophic medical expenditures and more out-of-pocket medical expenditures. No financial difference between southern and northern areas was observed, possibly because a similar proportion of the population was insured (85.6% vs 87.6%) and because had similar levels of economic development.

NHI was not found to effectively reduce regional inequality in medical financial burdens, but it was found to ease the financial burden of eastern residents more than it did for the northern residents, possibly because people residing in mountainous areas or on offshore islands are exempt from co-payment fees. Since the eastern area, including Taitung, Hualian, and Penghu^1 ^Counties, is less developed than other areas in Taiwan, the Bureau of NHI has tried to increase accessibility of medical care there, especially in the aboriginal areas and outlying islands, in many ways. With regard to supply, a number of medical payment incentives have been implemented to encourage healthcare providers to extend medical care to residents of those areas. These incentives include higher insurance reimbursed outpatient physician fees, no limit on the number of insurance-reimbursed visits a patient would make to a physician and increased payment for circuit medical services. With regard to demand, the Bureau of NHI exempts residents in these areas from co-payment requirements and covers the registration fees in aboriginal areas, making the cost of medical care there the lowest in Taiwan. Furthermore, for the people in remote areas, the Bureau of NHI subsidizes transportation costs, including emergency helicopter transportation.

## Conclusion

The implementation of NHI is thought to be the most important health care reform in Taiwan. It has not only increased accessibility of health care service, but it has also made impressive strides towards the reduction of out-of-pocket medical expenditures. Such an outstanding achievement in Taiwan's public health has been praised by both domestic residents and international health experts. Nonetheless, major improvements are still required to reduce the financial burden imposed on certain disadvantaged groups, such as the poor and aborigines. The time and money it takes to reach health facilities increases as the distance to these medical institutions increases. Such costs create a greater financial burden on socio-economically disadvantaged groups. In spite of free health services provided by NHI, it is likely that the cost of accessing health care negatively affects their decisions to receive medical assistance. Therefore, outreach services that target groups for whom access to health care remains problematic should be expanded under the auspices of universal health insurance.

Although the data in this study provides information on household consumption of health care, it only focuses on the collection of out-of-pocket health expenditures. This data is limited in that it does not allow us to further investigate any changes in health care use with the implementation of NHI. For example, we were not able to explore the differences in health care use, total health expenditures and estimates of elasticity of utilization among different income quintiles and regions in this paper. Future studies can focus on these issues using insurance claim data collected by the Bureau of NHI.

## Competing interests

The author(s) declare that they have no competing interests.

## Authors' contributions

TB, TC and CS contributed to conceptualization, analysis, and writing of the article. YW helped to perform the statistical analysis as well as to interpret findings. WT supervised all aspects of policy implications. All of us approved the final version. TC is the guarantor.

## Pre-publication history

The pre-publication history for this paper can be accessed here:


